# Virtual Visual Effect of Hospital Waiting Room on Pain Modulation in Healthy Subjects and Patients with Chronic Migraine

**DOI:** 10.1155/2013/515730

**Published:** 2013-01-10

**Authors:** Marina de Tommaso, Katia Ricci, Luigi Laneve, Nicola Savino, Vincenzo Antonaci, Paolo Livrea

**Affiliations:** ^1^Biomedical, Neuroscience and Sensory System Department, Bari Aldo Moro University, Policlinico General Hospital, Piazza Giulio Cesare, 11 70124 Bari, Italy; ^2^Automation and Control Unit, Informatic Engignery Division, Cetma Consortium Mesagne, Appia Way No. 7, 72100 Brindisi, Italy; ^3^Antonaci and Partner Studio, Mazzini Place, 73100 Lecce, Italy

## Abstract

Environmental context has an important impact on health and well being. We aimed to test the effects of a visual distraction induced by classical hospital waiting room (RH) versus an ideal room with a sea view (IH), both represented in virtual reality (VR), on subjective sensation and cortical responses induced by painful laser stimuli (LEPs) in healthy volunteers and patients with chronic migraine (CM). Sixteen CM and 16 controls underwent 62 channels LEPs from the right hand, during a fully immersive VR experience, where two types of waiting rooms were simulated. The RH simulated a classical hospital waiting room while the IH represented a room with sea viewing. CM patients showed a reduction of laser pain rating and vertex LEPs during the IH vision. The sLORETA analysis confirmed that in CM patients the two VR simulations induced a different modulation of bilateral parietal cortical areas (precuneus and superior parietal lobe), and superior frontal and cingulate girus, in respect to controls. The architectural context may interfere with pain perception, depending upon the status of subject. Many variables may change patients' outcome and support the use of VR technology to test the best conditions for their management.

## 1. Introduction

Pain is a complex function of the human brain, involving attention and emotions [1, 2]. The process of distraction appears to involve competition for attention between a highly salient sensation (pain) and consciously directed focus on some other information processing activity. In fact, the neuronal network, devoted to painful inputs elaboration, is not nociceptive specific, similar activations being produced by relevant tactile, auditory, and visual stimuli [3, 4]. The multisensory nature of this network makes it a likely candidate for cross-modal modulation of pain [5]. In this sense, growing interest exists toward virtual reality (VR) technology, which is based on integrating multimodal (visual, auditory, tactile, and olfactory) sensory distractions [6]. Virtual reality (VR) is as effective in inducing emotional responses as reality and its application is extremely valuable in exposure treatment [7]. In fact, in virtual environments, the patients experience similar physiological symptoms as they do in real life situations, so virtual models can be employed to test how the ambient of real life could possibly interfere with pain modulation. Presently there is a growing body of evidence on the impact of the environment on health and well-being, and some aspects of hospital rooms have been studied as “positive distracters” able to reduce patients experienced stress [8, 9]. However, few studies evaluated the effects of potential positive distracters as art or access to nature on pain as a measure of stress. In previous studies, we employed laser-evoked potentials (LEPs), a neurophysiological method specific for nociceptive pathways and cortical areas devoted to pain elaboration [10, 11] and observed that the aesthetic content of the visual stimuli is able to condition pain sensation in healthy subjects [12]. Also the emotional correlates of visual experience seemed to modify brain response to painful stimuli in both healthy subjects and migraine patients [13–15]. Migraine is a disorder of neurovascular origin, which may become chronic and invalidating. Distraction induced by cognitive task was ineffective in modulating pain processing in chronic migraine [16], differently from the vision of affective images which was able to reduce LEPs amplitude in the same type of patients [14]. 

 In this study, we aimed to test the effects of a visual distraction induced by classical hospital waiting room versus an ideal room with a sea view, both represented in virtual reality, on subjective sensation and cortical responses induced by painful laser stimuli in healthy volunteers and patients with chronic migraine.

## 2. Methods

### 2.1. Cases

 Sixteen consecutive outpatients, who received a diagnosis of chronic migraine (CM) at the Neurophysiopathology of Pain Center of Bari Policlinic General Hospital, were recruited for the study. They were 10 females and 6 males, 20–44 aging (mean age 34.3 ± 8.4), diagnosed in accord to the Headache Classification Committee [17]. The diagnosis was supported by a 3-month observation preceding the recording session. Patients with persisting CM after withdrawal of analgesics during the previous three months observation were considered for inclusion in the study. The mean migraine history duration was 20.1 ± 4.5 years, the mean headache frequency (average number of days/headache in a month computed in the last three months) was 19.9 ± 3.4 days. Sixteen nonmigraine controls (10 females, 20–43 aging mean age 33.9 ± 6.8 years) were also evaluated. Exclusion criteria were general medical, neurological, ocular, or psychiatric diseases. Females were recorded 14.23 ± 2.1 days after menses and no one assumed contraceptive therapies. All patients were in the interictal state, the time from the end of the last attack and symptomatic treatment-taking (triptans in all cases) being at least 24 hours, while an interval of at least 24 hours from the next attack was ascertained by a telephonic interview. Longer intervals were not allowed for the diagnosis of chronic migraine. In all patients anxiety, depression, severity of headache, and quality of life were evaluated by means, respectively, of self-rating anxiety (SAS) and depression scale (SDS) [18, 19], MIDAS questionnaire [20], and Short-Form 36 (SF-36) Health Survey [21].

EEG recording and laser stimulation. All subjects were recorded during the morning. They were generically advised that laser-evoked potentials (LEPs) would be performed during a virtual reality (VR) simulation, but that they should pay attention to stimulus intensity, in order to rate it after each VR session, using a visual analogical scale, where the absence of pain (value 0) was represented in white and the maximum pain (value 100) in intense red. No participant was informed about the content of the VR representation. All subjects gave their written consent to the study, which had been previously approved by the Ethic Committee of our Hospital. Each subject was seated in a comfortable chair positioned in a quiet room with an ambient temperature of 21°C to 23°C, in an awake and relaxed state. The pain stimulus was a laser pulse (wavelength 10.6 mm) generated by a CO^2^ laser (Neurolas, Electronic Engineering, Florence, Italy; http://www.elengroup.com/). The beam diameter was 2.5 mm and the duration of the stimulus pulse was 25 msec. Stimulus intensity was settled at each subject suprapain threshold, that is, at 2.5 Watt over the intensity level which determined a pinprick sensation after at least 10 of 20 ascending and descending test stimuli. The dorsum of the right hand was stimulated in all cases. Irradiated points differed slightly between each stimulus to minimize skin burn, nociceptor fatigue, and central habituation. Thirty laser stimuli were delivered during the each VR session, using a 10 sec interstimulus interval. 

EEG was recorded by 62 scalp electrodes, according to enlarged 10–20 system (Fp1, Fpz, Fp2, F7, F3, Fz, F4, F8, T3, C3, Cz, C4, T4, T5, P3, Pz, P4, T6, O1, Oz, O2, FC2, FC1, CP1,CP2, PO3, PO4, FC6, FC5, CP5, CP6, AF7, AF3, AFZ, AF4, AF8, F5, F1, F2, F6, FT7, FC3, FCZ, FC4, FT8, C5, C1, C2, C6, TP7, CP3, CPZ, CP4, TP8, P5, P1, P2, PO7, POZ, PO8) impedance below 5000 ohms, referring to the nasion with the ground at Fpz. Another electrode was placed above the right eye to record the EOG. Signals were amplified, filtered (0.5 to 80 Hz, at a sampling rate of 256 Hz), and stored on a biopotential analyzer (Micromed System Plus; Micromed, Mogliano Veneto, Italy; http://www.micromed-it.com/).

**Figure 1:**
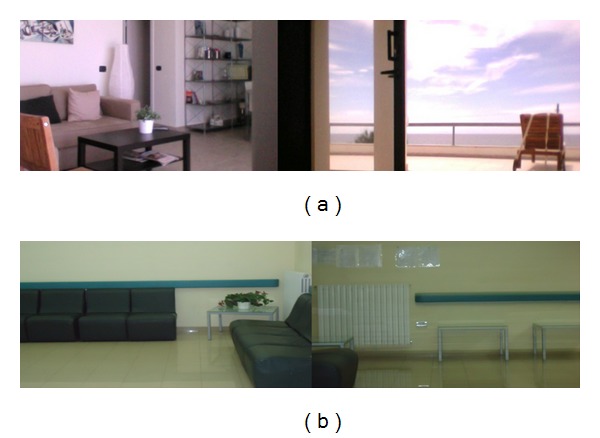
Two static images of our Virtual Reality representation. RH: Real Hospital, IH: Imaginary Hospital.

Virtual Reality employment and experimental procedure. The VR system was provided by the CETMA Consortium (http://www.cetma.it/). Subjects were engaged in a fully immersive VR experience through a head-mounted display (HMD), where two types of waiting rooms were simulated. The images changed allowing the subject to explore the entire virtual room and have the illusion of being completely surrounded by the virtual environment, without any vision of the laboratory. We directly evaluated the effect of virtual representation of two different simulated rooms. The Ideal Hospital (IH) represented a large room provided by an access to nature via a large window on the sea, natural luminosity, and sofa and furniture for houses use (Figure 1(a)). The Real Hospital (RH) simulated a classical waiting room, different from those of our hospital, characterized by comfortable but standard sofa with absence of natural light and windows (Figure 1(b)). The representation was settled to give the impression to be seated looking around the virtual room. The luminosity and color contrasts were exactly the same in the two conditions, though in the IH they were virtually provided by a natural source and in the RH by an artificial source of light. In fact, in the RH the virtual exploration of the room provided the vision of a wall of clear color, while in the IH condition subjects looked at a large window with sea and sky (Figure 1).

The RH and IH representations lasted at least 10 min each, and laser stimuli were delivered after about 3 min of adaptation to the virtual ambient. The RH and IH VR sessions were randomly presented across subjects. At this stage we avoided evaluating the VR impact on laser-evoked responses and proceeded with the comparison of the effects of the virtually represented visual contests. We also did not include a condition of “no-vision” (as a black panel) which seemed to us not fundamental in the evaluation of the impact of real life environment on pain feeling and elaboration. 

EEG analysis and statistical evaluation. All EEG were submitted to artifact detection, where signals similar for amplitude and phase to the ocular movement recorded over the EOG channels were discarded from LEPs averaging. For each subject, at least 15 artifacts free EEG traits with an epoch time of 1 s and 100 ms of prestimulus analysis were averaged. The sampling rate was 256 Hz. A digital filter in the bandpass 0.1–30 Hz was also applied. The average across the two VR conditions was done in any case employing ASA software vers 4.8. (ANT Software, Enschede, Netherlands; http://www.antneuro.com/). 

A preliminary EEG analysis was performed by an examiner blinded to the conditions (RH versus IH) and the groups (chronic migraine-CM—versus nonmigraine controls—C-) considering the CZ derivation, referred to nasion, and T3, referred to Fz. This montage allows evaluating the N2-P2 vertex components and the N1 temporal component features [11]. Latency and amplitudes were analyzed by means of Student's *t*-test for paired data, to compare LEPs in RH versus IH conditions in the single groups. A repeated measures ANOVA was also performed for LEPs considering the RH versus IH conditions as the within groups and the diagnosis CM versus C as the between groups factor. These analyses were performed by SPSS vers. 20. In migraine group the percent ratio of pain rating and N2-P2 amplitude in RH/IH conditions was correlated with frequency and severity of migraine, anxiety and depression scores, and quality of life, by means of Spearman correlation test. 

LEPs were further analyzed using standardized low resolution brain electromagnetic tomography (sLORETA) by the LORETA-KEY (publicly available free academic software at http://www.uzh.ch/keyinst/loreta.htm) [22–24] Numerous studies have supported the usefulness and validity of LORETA in localizing generators of scalp-recorded potentials, including recent research on pain processing and modulation, which have highlighted changes in cortical activity in areas of the pain matrix [25, 26]. 

For sLORETA, statistical differences between conditions were computed as images of voxel-by-voxel *t*-values. The localization of the differences in cortical activity was based on the standardized electric current density and resulted in 3-dimensional *t*-score images. In these images, cortical voxels of statistically significant differences were identified by a nonparametric approach with 5% probability level threshold determined by 5000 randomizations [22]. A randomization procedure was implemented to control for type I errors arising from multiple comparisons [27].

Firstly LEPs in RH versus IH conditions were compared in CM and C groups separately, employing the *t*-test for paired data (paired groups test RH = IH), then independent groups analysis, testing (RH-IH in CM group) = (RH-IH in C group), was carried out. The randomization procedure for the Statistical non-Parametric Maps (SnPM) was applied, with 5000 randomization, according to sLORETA software [22]. 

## 3. Results

All patients and controls confirmed that RH simulated a hospital waiting room and that IH represented a room with a nice sea view.

### 3.1. Pain Rating

The VAS values changed in opposite direction in the two groups, with an increase and oppositely a decrease in RH versus IH condition, respectively, in controls and in chronic migraine (Table 1). However, these changes did not reach the statistical significance in control group (*t*-value −1.95 DF 15 n.s.), while the paired *t*-test was significant in chronic migraine group (*t*-value 2.26 DF 15 *P* 0.039). The repeated measure ANOVA did not show significant changes within groups for the factor condition RH versus IH, while the interaction condition × group was significant (*F* = 8.32 DF 30 *P* 0.008) as well as the between group factor (Cm versus C *F* = 80.4  *P* < 0.0001).

### 3.2. Laser-Evoked Potentials

The latencies and amplitude of N1 component, computed on the T3-Fz derivation, were not significantly modified in the migraine and control groups in the RH versus IH condition (N1 latency in CM group *t*-test 0.38 n.s; N1 latency in control group, *t*-test 0.53 n.s. N1 amplitude in CM group, *t*-test −1.73 n.s, N1 amplitude in control group, *t*-test −0.93 n.s.). The repeated measures ANOVA did not give significant results for the within and between groups factors.

The P2 latency was slightly but not significantly reduced in CM group in the IH compared to the RH condition and no significant changes were observed in regard to N2 latency (N2 latency in CM group *t*-test 0.39 n.s.; P2 latency in CM group, *t*-test 2.67 n.s). In control group no significant N2 and P2 latencies changes occurred between the two VR presentations, though a slight P2 latency reduction was present in IH condition (N2 latency in control group *t*-test 0.38 n.s.; P2 latency in control group *t*-test 0.84 n.s.). The repeated measures ANOVA showed that both the P2 and N2 latencies did not change significantly for the condition within subjects (N2 *F* 2.3 DF 1 n.s, P2 *F* 1.8 n.s.), as well as for the interaction condition IH versus RH × diagnosis (N2 *F* 0.83 DF1 n.s, P2 *F* 3.72 *P* 0.06). The between groups factor (CM versus C) comparison did not provide significant results either for N2 or P2 latencies (N2 *F* 1.80 DF 1 n.s, P2 *F* 2.1 n.s) (Table 1).

**Figure 2:**
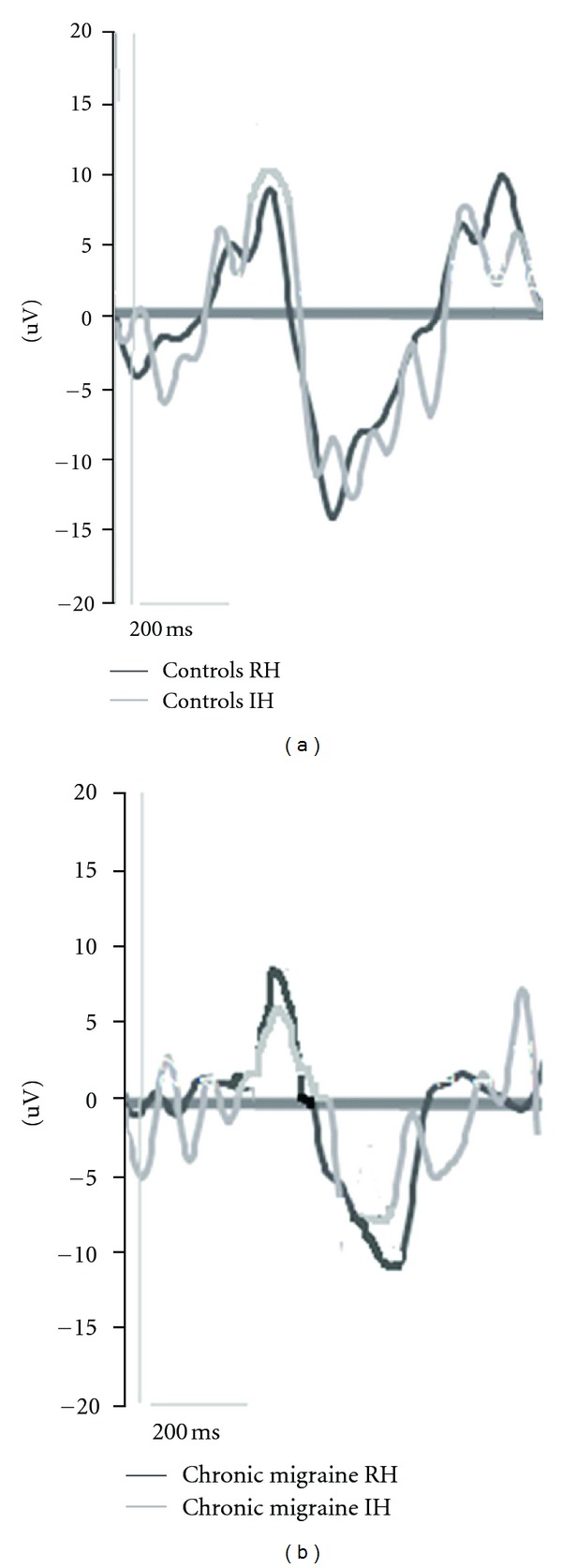
Grand average of laser-evoked potentials recorded at the vertex in 16 control subjects and 16 chronic migraine patients during Real Hospital (RH) and Imaginary Hospital (IH) virtual reality exposition.

Regarding the N2-P2 complex amplitude, it appeared significantly reduced in CM group during the IH simulation (N2-P2 amplitude in CM group, *t*-test 2.37 DF 17 *P* 0.029), while no significant changes were found in controls (N2-P2 amplitude in control group, *t*-test 0.41 n.s.). The repeated measures ANOVA showed that the within groups factor (i.e., the condition RH versus IH) did not produce significant results (*F*: 1.19 n.s.), while the interaction with the diagnosis was significant (condition × diagnosis *F*: 4.38 *P* 0.048). The between groups factor comparison (CM versus C) was also significant (*F* 7.5 DF 1 *P* 0.02) (Figure 2).

In migraine group, the percent ratio of N2-P2 amplitude in RH/IH conditions were negatively correlated with quality of life indexes (Spearman correlation test: SF36 Physical Component: −0.524 *P* 0.019; Mental Component: −0.647 *P* 0.003) and positively correlated with frequency of migraine (0.546 *P* 0.014) and MIDAS scores (0.532 *P* 0.013). Similar correlations were found for the percent ratio of pain rating in RH/IH condition, which showed a positive trend with frequency of migraine (0.570 *P* 0.011) and MIDAS score (0.544 *P* 0.013) and a negative trend with quality of life (Spearman correlation test: SF36 Physical Component: −0.554 *P* 0.013; Mental Component: −0.455 *P* 0.038).

### 3.3. sLORETA Analysis

Voxel-by-voxel *t*-values were computed in C and CM groups in the time interval of N2-P2 peaks (200–400 msec), which showed significant changes, as detailed above.

**Figure 3:**
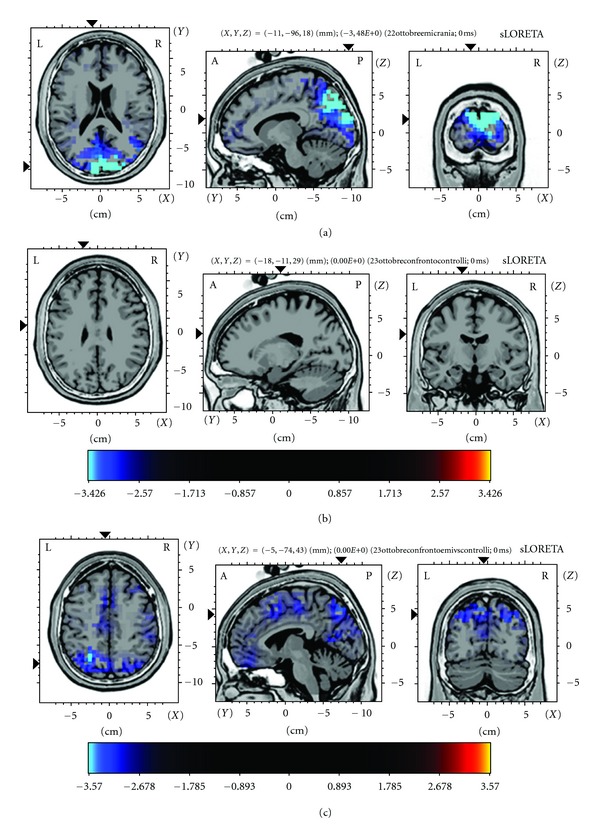
sLORETA Statistical Randomized Non-Parametric Maps showing locations of significant decreases and increases in brain activity during Real Hospital versus Ideal Hospital Virtual Reality Simulation in (a) chronic migraine patients (n°16) and (b) control group (n°16) (2-tailed paired *t*-tests). The critical threshold for statistical significance of *P* < 0.01 was automatically set to 3.42 (2.65 was the value for *P* < 0.05). The statistical comparison was done in the time interval 200–400 msec in both groups. A significant decrease of cortical activity was found in the bilateral precuneus (Broadman area 7) and cuneus (Broadman are 19) during the Real Hospital VR immersion. The statistical comparison (RH-IH in CM group) = (RH-IH in C group) is reported in (c). For this comparison, the critical threshold for statistical significance of *P* < 0.01 was automatically settled at 3.57 (2.64 was the value for *P* < 0.05). The bilateral precuneus (Broadman area 7) and medial frontal girus and anterior cingulate (Broadman area 32) were differently activated in patients and controls across the two VR conditions. The increased activation of these during the IH condition zones (or the reduced activation in RH one) separated significantly chronic migraine patients from controls.

In chronic migraine the comparison between the two conditions was significant for a reduced activation of occipital and parietal lobe in the RH condition (maximum difference on bilateral cuneus and precuneus—Broadman areas 7 and 19—), while the comparison between the two VR conditions did not cause significant changes in controls (Figures 3(a) and 3(b)). The statistical comparison (RH-IH in CM group) = (RH-IH in C group) confirmed that in chronic migraine the VR simulations induced a different modulation of bilateral parietal cortical areas (precuneus and superior parietal lobe, Broadman areas 7), and superior frontal girus and cingulate girus in area 32, in respect to controls (Figure 3(c)).

## 4. Discussion

In this study we evaluated for the first time the visual impact of different architectural contexts on an objective measure of pain perception, that is, the laser-evoked potentials, in healthy volunteers and a group of patients experiencing chronic migraine. This was obtained through a virtual reality simulation, which may itself modulate pain transmission and elaboration [6]. 

### 4.1. Pain Perception

The vision of the room with sea view was able to significantly reduce the subjective perception of laser pain in chronic migraine patients, differently from controls. In chronic migraine patients, the vision of different architectonical features was able to modulate pain perception, though in previous studies we observed a rigid pattern of pain modulation especially in the chronic form [28–30]. 

The interactions between visual and somatosensory systems are strong. In fact, concurring visual and painful stimulation may induce a form of “visual analgesia” [31], which may be based on a cognitive distraction from pain due to the contemporary engagement in stimuli encoding and rating and a distraction due to the interference with the emotional elaboration of nociceptive inputs. This latter form of distraction seems to be linked with the emotion-related arousal, as showed by studies based on both electrophysiological and neuroimaging measures, employing affective pictures vision during experimental pain [32, 33]. However, the possible analgesic effect of visual stimulation depends upon many variables, other than the subject of images, such as the modality of presentation. In the present study, we tried to simulate a condition of sitting and looking around a room. Neither healthy subjects nor controls had been previously advised on the content of the VR simulation, though the RH was clearly recognized as a hospital room. While normal subjects seemed not to be particularly influenced by the different content of VR immersion, except for a trend toward an increase of pain rating during the sea-room viewing, in patients the real hospital vision enhanced subjective rating of nociceptive stimuli. Considering that we did not consider the generic distracting effect of VR device, it was the hospital ambient that exerted this hyperalgesic effect on our chronic migraine patients. The other virtual room differed from the hospital one for an access to nature and furniture which are not usual in a conventional hospital ambient, though we cannot presently establish which visual element was able to interfere with pain perception. The present impression is that the visual impact of an architectural context may interfere with pain perception, depending upon the status of subject. In previous studies, pleasant or unpleasant pictures with high arousal were able to modulate pain perception [33]. Patients experiencing chronic pain may be negatively impressed by hospital vision, even if a comfortable waiting room is presented. It may suggest suffering, illness, and disability, with an effect similar to images showing human pain [32]. At this stage, we cannot exclude an aesthetic impact due to the beautiful sea view and nice furniture, but considering that normal subjects are susceptible to beautiful view [12], the contrast between the two environments was not probably perceived as ugly versus beautiful, but as hospital versus nonhospital context. This contrast may cause stronger arousal in chronic pain patients than in controls, modulating pain in a positive versus negative direction. 

The question if this increasing of pain feeling during hospital context viewing is peculiar of migraine, or if it may involve other types of chronic syndromes, may give aid to the new field of research on interventions to reduce environmental stressors through physical changes in hospital ambient. 

### 4.2. Laser-Evoked Potentials

In accord with subjective pain rating, CM patients showed an amplitude modulation of LEPs vertex complex during the VR simulation, with a decrease during the IH vision. In controls, no significant amplitude changes occurred between the considered experimental conditions. The N1 component, which refers to the earliest stage of perception processing [10, 34], was not involved by the changes occurring during the VR representation. Accordingly, N1 changes occurred neither during the contemporary vision of images with different affective content in migraine patients and controls, nor during the vision of beautiful pictures in healthy volunteers [12, 14]. The vertex complex was modulated in an increasing direction during the virtual hospital immersion in migraine, while a quite opposite trend was observed in healthy controls, in accord with the trend of pain rating. In this study we found a correspondence between N2-P2 vertex complex amplitude and pain rating, suggesting that the intensity of activation of nociceptive areas where these waves take origin correlates with perceived pain intensity. In fact, early and late LEP components are considered to be differentially sensitive to the subjective variability of pain perception: the late N2-P2 complex strongly correlates with perceived pain, whereas the early N1 component is thought to be a preperceptual sensory response [34]. Recently, Eck et al. employed FMRI and found that there is an involvement of brain regions associated with the affective and sensory-discriminative dimension of pain in the processing of pain-related words in migraine patients, and that the recruitment of those regions associated with pain-related affect is enhanced in patients with chronic pain experience [35]. Accordingly, the vision of the hospital may be perceived as “pain-related” in chronic migraine patients, while the room with sea viewing may be perceived as a reassuring and relaxing context, attracting patient's attention away from the laser stimulus. This type of attentive modulation may act on cortical areas devoted to the later stage of pain elaboration, which seem to generate the N2-P2 vertex complex [10]. Moreover, these waves seem not to be nociceptive specific, rather their magnitude correlates either with the subjective rating of saliency or with the arousal or attention reorientation to painful stimulus [4]. 

The different environments virtually represented were able to change the perceived relevance of painful stimuli in chronic migraine patients, the real hospital room increasing and the ideal room reducing the orienting response toward painful laser stimuli. If we accept that in virtual environments, the patients experience similar physiological symptoms as they do in real life situations [7], we can suppose that the different visual environmental contexts may exert a positive or negative influence on the cortical associative areas responsible of multimodal stimuli elaboration and pain feeling, depending on subject's status and personal experiences. An additional evaluation in episodic migraine would also further clarify if the present pattern is linked with migraine, or to the experience of chronic illness, also if this latter hypothesis may be presently supported by the correlation between the increment of pain perception during the real hospital viewing and the severity of migraine and poor quality of life. 

*sLORETA Analysis*—sLORETA analysis has been recently employed in research on pain processing and modulation [26, 36]. Substantial congruence has been demonstrated between LORETA and FMRI localization [37]. Cortical areas subtending the LEPs vertex complex were found to change the degree of activation during distraction tasks. In particular, in a previous study the insula showed a lower level of activation during a visual cognitive task contrasting painful laser stimulation [26]. This was explained by a reduced capacity in pain encoding for a concurrent cognitive employment.

In our study, the posterior cortical zones located in the cuneus e precunes seemed differently activated during Real Hospital and Ideal hospital viewing in chronic migraine patients. These cortical zones seem devoted to the integration of visual experience with other multimodal stimuli. In FMRI experiments employed by contrasting painful with visual stimuli, the parietooccipital cortex activation was associated with a sort of visual analgesia [31, 32]. In our patients the reduced amplitude of the LEPs complex at the vertex during the Ideal hospital viewing, which corresponded to a decrease of pain rating, was associated to a major diffusion of this activity on the parietooccipital zones. This may imply that during the virtual immersion in a luminous room very dissimilar to a classical hospital ambient, the visual associative zones may contribute to modulate pain elaboration probably reducing the recruitment of those areas which seemed very active in the noxious stimuli processing in chronic migraine [38]. Moreover, the distraction from pain is subtended by different cortical areas, depending from the type of concurrent task [26]. We also observed that other cortical zones in the medial frontal girus and anterior cingulate showed an increased activation during the virtual immersion in the room with the sea-view, and that the different recruitment of these areas during the tasks separated migraine patients from controls. These results may confirm that in patients with chronic migraine the visual stimulation induced by environmental context is able to modulate pain perception, activating cortical associative areas which integrated visual stimuli with nociceptive inputs and emotional experiences. In fact, in a previous study where painful laser stimulation was contrasted by the vision of affective images [14], LEPs appeared to be modulated in migraine patients, suggesting an active interaction between visual and somatosensory systems, which may be taken into account in the clinical management of migraine patients. At this stage we cannot establish which element of visual stimulation was able to interact with pain perception. Though the virtual immersion was characterized by identical physical features in terms of luminosity and contrast, it is presently difficult to establish which element of the images was able to interact with pain perception, if the access to nature by the window, or in general the dissimilarity from the hospital context.

*Final Comments*. Our study showed that the virtual environment may interfere with pain perception in chronic migraine patients. These results may give aid to further research on those factors which may potentially influence cognitive and emotional aspects of patients' experience, as the “positive or negative distracters” present in the environment. So far many variables may change patients' outcome and support the use of VR technology to test the best conditions for their management. 

## Figures and Tables

**Table 1:** Mean (M) and Standard Deviation (SD) values of subjective perception of laser pain measure by 0–100 Visual Analogic Scale (VAS) and latency and amplitude of laser-evoked potentials in Real Hospital (RH) and Imaginary Hospital (IH) virtual representation conditions.

			VAS	N1 Latency msec	N1 Amplitude uV	N2 Latency msec	P2 Latency msec	N2/P2 Amplitude uV
Controls N° 16	RH	M	30.5	169.45	6.31	216.37	321.87	24.58
		SD	13.5	43.40	3.78	29	85	9.48
	IH	M	34.5	162.45	7.71	219.32	308.12	26
		SD	18.5	39.97	3.58	29.9	45	16.08

Chronic migraine N° 16	RH	M	31.5	174.75	4.16	248.11	366	20,27
		SD	23.3	21.5	2.31	45.11	37.7	8
	IH	M	29.3	167.5	5.99	225.22	338.1	14,24
		SD	22.3	39.97	4.33	42.45	32.2	6.6
